# Development and validation of a brief digital cognitive test based on the paradigm of stimulus equivalence in a sample of older adults

**DOI:** 10.1590/1980-5764-DN-2022-0050

**Published:** 2023-12-04

**Authors:** Marcos Hortes Nisihara Chagas, Andreza Gomes Spiller Nery, Ana Julia de Lima Bomfim, Natalia Mario Aggio

**Affiliations:** 1 Universidade Federal de São Carlos, Grupo de Pesquisas em Saúde Mental, Cognição e Envelhecimento, São Carlos SP, Brazil.; 2 Universidade de São Paulo, Programa de Pós-Graduação em Saúde Mental, Ribeirão Preto SP, Brazil.; 3 Universidade de Brasília, Departamento de Processos Psicológicos Básicos, Brasília DF, Brazil.

**Keywords:** Aged, Cognition, Neuropsychological Tests, Learning, Idoso, Cognição, Testes Neuropsicológicos, Aprendizagem

## Abstract

With the technological advancement and democratization of electronic devices, computerized cognitive tests have been increasingly used in the clinical context to evaluate cognitive performance in individuals.

**Objective:**

This study aimed to propose a brief digital cognitive test based on the paradigm of stimulus equivalence and assess its convergent validity by comparing it with traditionally applied tests.

**Methods:**

The study was carried out with a non-probabilistic sample of 50 older adults selected from a public call through the communication media, health units, and day centers of a city in the countryside of São Paulo. Participants were assessed by the brief digital cognitive test, Mini-Mental State Examination, Brief Cognitive Screening Battery, and the Five Digit Test.

**Results:**

Participants had a mean age of 71.23 years (standard deviation [SD]: ±9.36) and a mean of 7.15 years of schooling (SD: ±5.34). The mean time to answer the test was 5.33 minutes (SD: ±1.92). There were statistically significant correlations between traditional and digital tests in most domains evaluated. In addition, considering the total score of the digital test, the test could discriminate participants with and without cognitive impairment: area under the ROC curve=0.765; 95%CI 0.630–0.901.

**Conclusion:**

The brief digital cognitive test, using the stimulus equivalence paradigm, is an easy-to-apply and valid instrument for the investigation of cognitive impairment in older adults.

## RESUMO

Com o avanço tecnológico e a democratização de dispositivos eletrônicos, os testes cognitivos computadorizados vêm sendo cada vez mais utilizados no contexto clínico para avaliar o desempenho cognitivo de indivíduos.

**Objetivo:**

Este estudo teve como objetivo propor um Teste Cognitivo Digital Breve baseado no paradigma de equivalência de estímulos e avaliar sua validade convergente comparando-o com testes aplicados tradicionalmente.

**Métodos:**

O estudo foi realizado com uma amostra não probabilística de 50 idosos selecionados por chamada pública pelos meios de comunicação, unidades de saúde e centros dia de uma cidade do interior de São Paulo. Os participantes foram avaliados pelo Teste Cognitivo Digital Breve, Miniexame do Estado Mental, Bateria Breve de Rastreio Cognitivo e Teste dos Cinco Dígitos.

**Resultados:**

Os participantes tinham média de idade de 71,23 anos (desvio padrão — DP: ±9,36) e média de anos de estudo de 7,15 anos (DP: ±5,34). O tempo médio para a realização do teste foi de 5,33 minutos (DP: ±1,92). Houve correlações estatisticamente significativas entre os testes tradicionais e o Teste Digital na maioria dos domínios avaliados. Além disso, considerando o escore total do teste digital, o teste foi capaz de discriminar participantes com e sem comprometimento: área sob a curva ROC=0,765; IC95% 0,630–0,901.

**Conclusão:**

O teste digital cognitivo breve com o uso do paradigma de equivalência de estímulos é um instrumento de fácil aplicação e válido para a investigação de comprometimento cognitivo em idosos.

## INTRODUCTION

Cognition is the ability to acquire and process knowledge and is commonly divided into domains for assessing and categorizing individual cognitive performance^1^. Cognitive performance gains special importance during the aging process, considering the natural and pathological decline that can occur over the years^2^.

Population aging is a worldwide phenomenon characterized by changes in the age pyramid. In view of this demographic scenario, investigating cognitive changes in this population becomes of the utmost importance. Cognitive assessment is traditionally performed with pen and paper; therefore, the available and validated tests are conducted in this way^3^. With the technological advancement, smartphones and tablets, computerized and digital cognitive tests are increasingly used in the clinical context^4–6^. The advantages of using computerized cognitive tests include the possibility of being self-applied with standardization, the chance to assess a greater number of people, and obtain greater data accuracy according to the test^6^. On the other hand, the individual subjected to the test would need skills for handling electronic devices^7^.

In the elaboration of computerized tests, different approaches can be used, such as the adaptation of a traditional test with minimal changes in its structure^8,9^, the development of a test based on an existing one, or from a paradigm other than the existing ones. A paradigm widely used in learning semantic relations with children and adults with intellectual and cognitive difficulties is stimulus equivalence.

When formalizing the stimulus equivalence paradigm, Sidman and Tailby proposed that symbolic and meaning relations can be operationalized from the verification that the stimuli involved in the class have reflexivity, symmetry, and transitivity properties^10^. Reflexivity is observed when, from training the relation between two stimuli, for example between A and B, the participant is able to respond to identical stimuli without direct training (AàA and BàB). In symmetry, when learning the AB relation, the inverse relation emerges (if AàB, so BàA). Finally, transitivity refers to the emergence of relations between stimuli that were never directly trained (if AàB and AàC, then BàC and CàB)^11,12^. One way of forming the equivalence classes is through the matching-to-sample (MTS) procedure, in which the relations between the arbitrary stimuli are taught^10^.

Various studies that evaluated this paradigm to assist in the learning of individuals with cognitive difficulties developed tasks for the formation of equivalence classes with the aid of electronic devices^13,14^. These studies also pointed out difficulties in forming equivalence classes in aged individuals with cognitive impairment and a possible positive correlation between performance in class formation and performance in cognitive assessment tests^15,16^. Thus, this study aims to propose a brief digital cognitive test based on the stimulus equivalence paradigm, comparing it with traditionally applied tests.

## METHODS

### Participants

The study was conducted with a non-probabilistic sample composed of 50 older adults. The inclusion criterion was age 60 years or older, and the exclusion criteria were the presence of severe visual or auditory deficits that would hinder the understanding of the tests, the presence of severe clinical or psychiatric comorbidity that would prevent conducting the tests, and self-reported lack of skill with electronic devices. These criteria were verified according to the researchers’ evaluation and based on the volunteers’ reports.

### Cognitive assessment

### Mini-mental state examination

The mini-mental state examination (MMSE) is an instrument widely used to screen for cognitive impairment that assesses cognition in a general manner. Its total score can vary from 0 to 30 points, and the lower the score, the greater the cognitive impairment^17^. In our study, the cutoff points proposed by Almeida^18^ were used to separate the participants into groups with and without cognitive impairment, being 19/20 for older adults with no schooling and 23/24 for those with previous school history^18^. The MMSE was used as a measure of general cognition.

### Brief cognitive screening battery

The brief cognitive screening battery (BCSB) was developed by Nitrini et al.^19^ with the objective of obtaining an instrument with less influence from schooling. It includes the figure memory test (incidental, immediate, learning, delayed, and recognition), verbal fluency (animals), and clock-drawing test^19^. The accuracy to differentiate healthy older adults from those with great neurocognitive disorder was previously tested in the Brazilian population^20^. In our study, we used the sum of the correct answers of the list of figures as indicative of immediate memory, the correct answers of the list of figures after verbal fluency and clock-drawing as late memory, the verbal fluency of animals as language, and the clock-drawing as executive functioning.

### Five digit test

The Five digit test (FDT) was developed by Sedó^21^ and consists of four stages: reading, counting, choice, and alternation. In the reading stage, the numbers must be read as fast as possible. In the counting stage, individuals are instructed to count the asterisks. In choosing, the participants are instructed to count the numbers that appear in the figure and they must inhibit the reading of the numbers. Finally, in the alternation phase, it is necessary for the participant to count how many numbers are in the quadrants, except in the quadrants with darker edges, which are requested to be read. The time of each stage is measured, providing measures of processing speed (reading and counting), selective attention/inhibitory control (choice), and alternating attention/cognitive flexibility (alternation)^22^. In our study, we used the sum of reading and counting as a measure of processing speed, the time of the counting stage as a measure of selective attention, and the time of the alternation stage as a measure of alternating attention.

### Brief digital cognitive test

The brief digital cognitive test (BDCT) was elaborated by the researchers according to the paradigm of stimulus equivalence, using OpenSesame 3.1 software^23^. The test consisted of two classes of stimuli with three stimuli in each class. Thus, Class 1 was composed of a house (stimuli A1), a square (B1), and one abstract figure (C1), while Class 2 was composed of a tree (A2), a triangle (B2), and another abstract figure (C2). The test was constructed with seven blocks (Table 1).

**Table 1:** Sequence of the task’s blocks, acronym of the block name, trained or tested relations in each block, presence of feedback, and number of trials in each block.

Block	Procedure	Relations	Feedback	Total trials
	Instruction			
1	AA Training	AA	Yes	12
	Instruction			
2	AB baseline training	AB	Yes	12
	Instruction			
3	ABBA baseline and symmetry test	AB and BA	No	8
	Instruction			
4	AC baseline training	AC	Yes	12
	Instruction			
5	ACCA baseline and symmetry testing	AC and CA	No	8
6	ABBA baseline and symmetry test	AB and BA	No	8
7	BCCB transitivity test	BC and CB	No	8

The training and test trials were made through simultaneous MTS trials, in which the model and comparison stimuli appeared on the screen at the same time. The screen of the portable computer was divided into quadrants so that in the middle of the two upper quadrants was the model stimulus and, in the lower quadrants, the two comparison models. In all blocks, the comparison stimuli were randomized to appear half of the time in the lower right-hand quadrant and the other half in the lower left-hand quadrant. In addition, the presentation of the relationships in each trial was randomized within each block. The choice of the comparison stimulus was made by touching the computer screen on the quadrant of the stimulus.

In the training blocks, the answers defined as correct produced a green “V” sign on the screen, and the answers defined as incorrect produced a red “X” sign. The duration of the feedback screen was 750 milliseconds. There was no feedback in the test blocks. Between each trial, a white screen appeared for 500 milliseconds.

At the beginning of the procedure, the participant was positioned in front of the screen of a portable computer and was instructed to perform the test according to the guidelines that would appear on this screen. Before each block, instructions were given to the participants, with the same characteristics, explaining the task to be performed and the presence or not of feedback.

**Figure 1:**
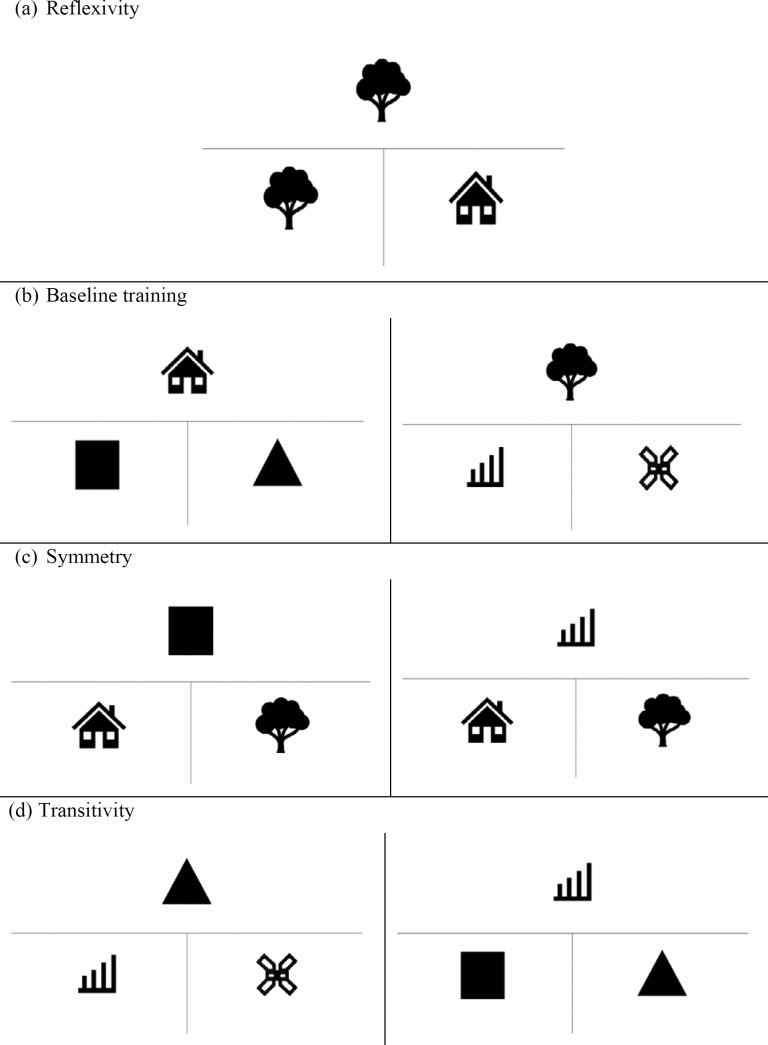
Examples of screens of the relations present in the blocks: (a) reflexivity (AA); (b) baseline training (AB and AC); (c) symmetry (BA and CA); (d) transitivity (BC and CB).

The following variables of the brief digital cognitive test were considered in the study:

- Total number of correct answers: composed of the sum of correct answers in the seven blocks;
- Reflexivity: correct answers for the AA block;
- Baseline training: sum of the correct answers for blocks AB and AC;
- Symmetry: sum of the correct answers in the BA and CA relations;
- Baseline test: sum of the correct answers for AB and AC;
- Late baseline and symmetry: correct answers for blocks AB and BA;
- Transitivity: correct answers for blocks BC and CB; and
- Total test time (Figure 1).

### Procedures

The participants were initially evaluated using a battery of traditional cognitive tests. Subsequently, the BDCT was applied through a portable computer with a 14-inch touchscreen. The assessment was performed at the participants’ homes or in health or social centers in an appropriate room, according to the participants’ preferences. The complete assessment lasted a mean of 60 minutes and was conducted individually. All volunteers consented to their participation by signing the Free and Informed Consent Form, which was approved by the Research Ethics Committee of the Federal University of São Carlos (CAAE: 12418019.0.0000.5504).

### Data analysis

Descriptive analysis was performed to characterize the sociodemographic profile of the groups. The Kolmogorov-Smirnov test was applied to verify data normality. The differences between the groups with and without cognitive impairment were assessed by the Mann-Whitney test. The Spearman’s Correlation test was used to assess the correlation between the variables. The partial correlations were calculated to adjust the correlations by age and years of schooling. The area under the receiver operating characteristic (ROC) curve and the sensitivity and specificity for the best cutoff point of the total score of the digital test were calculated to discriminate between individuals with and without cognitive impairment. The best cutoff point was determined by the highest sum of sensitivity and specificity. The statistical analyses were performed using the Statistical Package for Social Sciences (SPSS), version 23.0. The level of significance considered was p<0.05.

## RESULTS

### Sociodemographic characteristics

The mean age of the participants was 71.23 years old (SD: ±9.36), with a median of 71 years old, varying from 60 to 100 years. Regarding the schooling level, 60% of the participants had between 0 and 4 years of study, 32% had between 5 and 8 years, and only 4 had more than 8 years. The mean schooling time was 7.15 years (SD: ±5.34), with a median of 4.5 years, varying between 0 and 18 years. Furthermore, 58% (n=29) were female and 60% (n=30) had no partner, belonging to the group of single, widowed, or divorced individuals.

### Performance in the brief digital cognitive test

Of the 50 participants, 32 (64%) always clicked on the answer area and 45 (90%) clicked twice or fewer times outside the answer area. The mean of total correct answers in the test was 51.34 (SD: ±10.02), varying between 35 and 68, which corresponds to a mean rate of correct answers of 75.5%. The mean time to answer the test was 5.33 minutes (SD: ±1.92), varying from 2.43 to 9.34 minutes. Three older adults clicked outside the lower quadrants in more than 10% of the trials, so they were excluded from the inferential analyses. Table 2 presents the means and medians for each variable of the BDCT.

**Table 2:** Measures of central tendency, standard deviation and interquartile range of the variables extracted from the brief digital cognitive test.

	Mean	SD	Median	IQR
Reflexivity	11.91	±1.56	12	1
Baseline training	17.77	±4.17	18	8
Baseline test	5.70	±1.94	6	3
Symmetry	5.47	±1.92	5	3
Late BL and symmetry	6.19	±1.83	7	4
Transitivity	5.06	±2.04	5	3
Total score	51.34	±10.02	52	19

Abbreviations: SD, standard deviation; IQR, interquartile range; BL, baseline.

Concerning the transitivity block, it was observed that only seven older adults correctly answered 100% of this block and six answered incorrectly in only one trial. Considering that these participants formed equivalence classes, only 27.65% of the sample managed to form such classes.

### Relation between age and schooling and variables of the brief digital cognitive test

Age showed a moderate negative correlation with the scores of the baseline training variable (r=-0.517; p<0.001), and a weak negative correlation with the scores of the variables: reflexivity (r=-0.355; p=0.014); symmetry (r=-0.384; p=0.008); transitivity (r=-0.360; p=0.013), and total score (r=-0.482; p=0.001). Conversely, it presented a weak positive correlation with total time (r=0.499; p<0.001).

In relation to years of schooling, there was a moderate positive correlation with the following variables: baseline training (r=0.575; p<0.001), total score (r=0.542; p<0.001), and total time (r=-0.530, p< 0.001). In addition, there was a weak positive correlation with the correct answers to reflexivity (r=0.462; p=0.001); symmetry (r=0.365; p=0.012); late baseline and symmetry (r=0.347; p=0.017), and transitivity (r=0.366; p=0.011).

### Correlations between the cognitive domains and variables of the brief digital cognitive test

Table 3 shows Spearman’s correlations between the BDCT and cognitive variables.

**Table 3:** Spearman’s correlation between general cognition and cognitive domains and variables of the brief digital cognitive test.

	General cognition	Immediate memory	Late memory	Language	Executive functioning	Processing speed	Selective attention	Alternated attention
Reflexivity	r=0.495 p<0.001	r=0.386 p=0.007	r=0.298 p=0.042	r=0.456 p=0.001	r=0.503 p<0.001	r=-0.486 p=0.001	r=-0.409 p=0.004	r=-0.496 p<0.001
Baseline training	r=0.510 p<0.001	r=0.573 p<0.001	r=0.560 p<0.001	r=0.552 p<0.001	r=0.424 p=0.003	r=-0.424 p=0.003	r=-0.354 p=0.015	r=-0.341 p=0.019
Baseline test	r=0.401 p=0.005	r=0.444 p=0.002	r=0.384 p=0.008	r=0.479 p=0.001	r=0.239 p=0.106	r=-0.401 p=0.005	r=-0.252 p=0.087	r=-0.305 p=0.037
Symmetry	r=0.408 p=0.004	r=0.505 p<0.001	r=0.423 p=0.003	r=0.366 p=0.011	r=0.243 p=0.100	r=-0.463 p=0.001	r=-0.475 p=0.001	r=-0.395 p=0.006
Late baseline and symmetry – test	r=0.509 p<0.001	r=0.213 p=0.151	r=0.230 p=0.120	r=0.368 p=0.011	r=0.252 p=0.087	r=-0.446 p=0.002	r=-0.399 p=0.005	r=-0.415 p=0.004
Transitivity	r=0.443 p=0.002	r=0.369 p=0.011	r=0.464 p=0.001	r=0.349 p=0.016	r=0.343 p=0.018	r=-0.409 p=0.004	r=-0.414 p=0.004	r=-0.424 p=0.003
Total score	r=0.591 p<0.001	r=0.577 p<0.001	r=0.544 p<0.001	r=0.593 p<0.001	r=0.451 p=0.001	r=-0.564 p<0.001	r=-0.504 p<0.001	r=-0.516 p<0.001
Total time	r=-0.550 p<0.001	r=-0.623 p<0.001	r=-0.597 p<0.001	r=-0.633 p<0.001	r=-0.469 p=0.001	r=0.638 p<0.001	r=0.548 p<0.001	r=0.487 p=0.001

Table 4 shows the partial correlations between the BDCT and cognitive variables adjusted by age and schooling years. All correlations between the total score of BDCT and the scores of general cognitions and domains remain statistically significant.

**Table 4:** Partial correlations between general cognition and cognitive domains and variables of the brief digital cognitive test adjusted by age and years of schooling.

	General cognition	Immediate memory	Late memory	Language	Executive functioning	Processing speed	Selective attention	Alternated attention
Reflexivity	r=0.189 p=0.099	r=0.137 p=0.177	r=0.108 p=0.233	r=0.084 p=0.285	**r=0.308** **p=0.017**	**r=-0.249** **p=0.044**	**r=-0.326** **p=0.012**	**r=-0.426** **p=0.001**
Baseline training	**r=0.326** **p=0.012**	**r=0.365** **p=0.005**	**r=0.329** **p=0.011**	r=0.207 p=0.079	r=0.203 p=0.084	r=-0.140 p=0.171	**r=-0.306** **p=0.017**	r=-0.194 p=0.093
Baseline test	**r=0.324** **p=0.012**	**r=0.316** **p=0.014**	r=0.210 p=0.076	**r=0.325** **p=0.012**	r=0.176 p=0.116	**r=-0.323** **p=0.012**	r=-0.158 p=0.141	r=-0.206 p=0.081
Symmetry	**r=0.315** **p=0.015**	**r=0.402** **p=0.002**	**r=0.285** **p=0.025**	r=0.096 p=0.258	r=0.075 p=0.306	**r=-0.310** **p=0.016**	**r=-0.305** **p=0.018**	**r=-0.260** **p=0.037**
Late Baseline and symmetry – test	r=0.431 p=0.001	r=0.077 p=0.301	r=0.108 p=0.233	r=0.121 p=0.207	r=0.126 p=0.197	r=-0.225 p=0.062	**r=-0.284** **p=0.025**	**r=-0.323** **p=0.013**
Transitivity	**r=0.271** **p=0.031**	r=0.158 p=0.141	**r=0.291** **p=0.023**	r=0.161 p=0.138	r=0.142 p=0.168	r=-0.019 p=0.448	**r=-0.281** **p=0.027**	**r=-0.279** **p=0.027**
Total score	r=0.459 p=0.001	r=0.382 p=0.004	r=0.351 p=0.007	r=0.259 p=0.038	r=0.260 p=0.037	r=-0.289 p=0.023	r=-0.405 p=0.002	r=-0.394 p=0.003
Total time	**r=-0.367** **p=0.005**	**r=-0.496** **p<0.001**	**r=-0.501** **p<0.001**	**r=-0.358** **p=0.006**	**r=-0.242** **p=0.049**	r=0.123 p=0.202	**r=0.255** **p=0.040**	r=0.117 p=0.214

Notes: Numbers in bold: correlations with p-values<0.05.

### Variables of the brief digital cognitive test divided by groups with and without cognitive impairment

Table 5 presents the differences between the scores of the participants with and without cognitive impairment groups according to the MMSE scores. There was no difference between groups for age (p=0.118).

**Table 5:** Median, interquartile range and differences between the groups with and without cognitive impairment.

	With cognitive impairment (n=25)		Without cognitive impairment (n=22)		p-value
	Median	25–75	Median	25–75	
Reflexivity	11	10–12	12	12–12	0.003
BL – training	15	13–21	21	17.75–21.25	0.011
BL – test	5	4.0–6.5	7	5–8	0.022
Symmetry	5	3.5–7.0	7	4–8	0.021
Late BL/S	5	4–7	8	6–8	0.003
Transitivity	4	3.5–6.0	6	4.00–7.25	0.080
Total score	45	38.5–55.0	57.5	51.00–62.25	0.002
Total time	5.81	4.48–7.38	4.10	3.14–5.61	0.008

Abbreviations: BL, baseline; BL/S, baseline and symmetry. Notes: 25–75: percentile 25% to percentile 75%.

**Figure 2:**
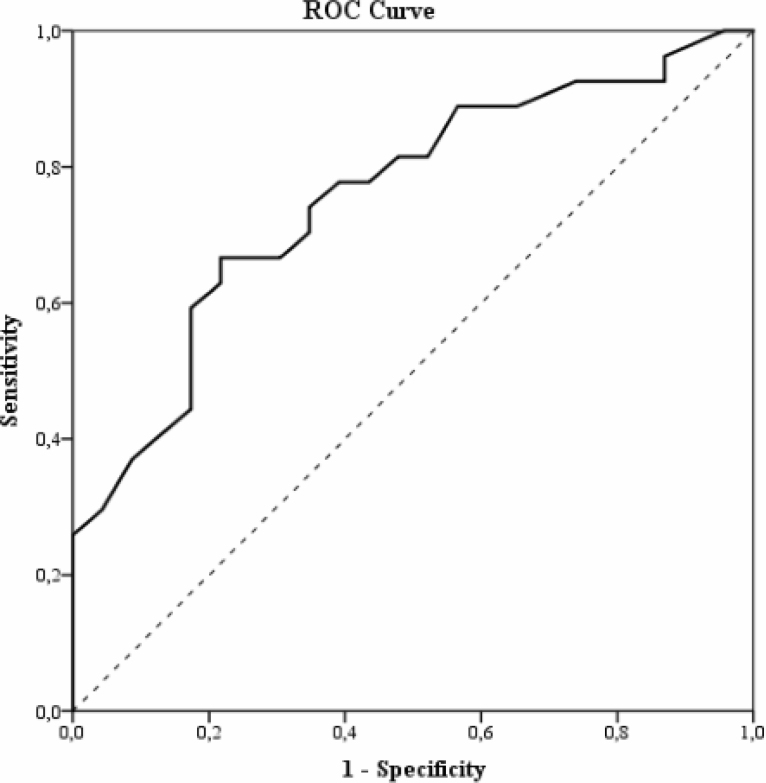
The receiver operating characteristic curve of the total score of the brief digital cognitive test to discriminate participants with and without cognitive impairment according to the mini-mental state examination score.

Abbreviations: ROC, receiver operating characteristic curve.

Considering the total score of the BDCT, the area under the ROC curve (Figure 2) was 0.765; 95% confidence interval (CI) 0.630–0.901, with the best cutoff point to discriminate participants with and without cognitive impairment being 53/54. At this cutoff point, sensitivity was 72%, and specificity was 68.2%.

## DISCUSSION

Most of the correlations between the variables of the BDCT and the scores of the traditional cognitive tests were statistically significant. Moreover, there were significant differences between the scores of older adults with and without cognitive impairment.

Regarding the mechanics of the test, it was observed that only five participants clicked more than twice outside the answer area, which can be an indication that the majority understood the test. It is worth noting that the test presented always uses tasks with the same pattern (matching-to-sample), differently from other digital or computerized cognitive tests^24–26^ that use different strategies to test cognition. One possibility that can be tested in the future would be not to allow the transition to the next trial if the participant clicks outside the answer quadrants.

The mean duration of the test was approximately five minutes, which is compatible with the brief and screening tests most used in our country^17,16^. The MMSE has a similar mean application time, while the Montreal Cognitive Assessment (MoCA) is applied in 5 to 10 minutes^17,26^. However, these traditional tests are migrating to versions requiring the payment of a fee for use^27^.

Concerning age and schooling, it was observed that there was a correlation between the BDCT scores and age and schooling, meaning that the higher the age, the lower the scores, and the higher the schooling, the higher the test scores. These findings are similar to those found in most traditional and computerized cognitive tests^28–31^. One aspect that must be considered is that age and schooling are also related to difficulties in operating digital devices^7^.

The study also evaluated the correlations between the test scores on paper and each stage of the BDCT. The ABBA block was the least correlated with the results of the traditional tests, having only correlated with the results of the MMSE and the FDT. This block was added as a trial to assess the memory of the first block, which was not observed in the results, since there was no correlation with the memory scores. Two possibilities must be considered for future adjustments to the test:

- Removing this block; or
- Including an instruction before this block since, in the test presented, the last three blocks have only one instruction, as shown in Table 1.

Another aspect observed was that the clock-drawing test may not be the most adequate to assess executive functioning in the sample of this study since this domain showed no correlation with three measures of the BDCT. Our sample included many participants with low schooling and it is known that the clock-drawing test presents limitations for assessing cognition in these populations^32,33^. Another possibility would be that the tasks of forming equivalence classes involve more processes of memory, language, and attention than executive functioning. The moderate correlation found between this test and the reflexivity block must also be emphasized, pointing to a possible need for abstraction capacity to understand the mechanics of the BDCT.

In the correlation table, it was also possible to notice that the correlations between baseline training and traditional tests were greater than those between baseline and traditional tests, which can be referred to two main factors:

- The number of trials in each block — while the baseline tests consisted of eight trials in total, the baseline training consisted of 24 trials; and
- The presence of feedback — this facilitates the understanding of the task and possibly leads to the use of fewer cognitive resources.

However, these possibilities must be evaluated more systematically in the future.

Additionally, the baseline training blocks were the most correlated with the memory domains. This points out that these domains are essential in the formation of equivalence classes since, without baseline learning, the entire process will be compromised.

In relation to the total BDCT score, a moderate correlation was observed with all scores of the traditional tests, especially the correlations found with general cognition and language, except for the clock-drawing. The language test used in this study deals with the categorical verbal fluency of animals; therefore, it is a task that assesses the participant’s categorization ability more than the language itself. It is also known that the tasks of forming equivalence classes are closely related to categorization ability. The results also corroborate previous studies that pointed to the correlation between the general results in the MMSE and the formation of equivalence classes^21^.

Although this study did not evaluate the diagnosis of each participant in relation to the diagnosis of mild or greater neurocognitive disorder, individuals were divided into two groups in order to determine the test’s ability to discriminate between subjects with and without cognitive impairment. It can be observed that there were differences between the groups for all stages of the digital test, except for transitivity, indicating that the test could be used in future research studies to evaluate its properties in clinical samples. Likewise, the area under the ROC curve is similar to that found in studies with other computerized cognitive tests validated to separate individuals with and without dementia^8,30,32^. In general, the sensitivity and specificity results were slightly lower when compared to other studies with traditional screening tests, although the values vary significantly across the studies^3^. However, it must be considered that we used a small sample compared to other validation studies.

An important limitation of our study is its non-probabilistic sample; therefore, it is possible that individuals who did not have ease handling electronic devices may not have volunteered for the research. Despite this, individuals with different schooling levels were included. In addition to that, as a criterion of cognitive impairment, we only used the MMSE cutoff points and the clinical diagnosis could be more reliable to separate these groups and determine discriminant validity. Despite this, we must emphasize that the BDCT proposal is easy to apply and can be available on different platforms, considering its extremely simple dynamics.

In conclusion, the BDCT with the use of the paradigm of stimulus equivalence is an easy-to-apply and valid instrument for the investigation of cognitive impairment in older adults. Future studies must be conducted to analyze different arrangements of instruments using this paradigm such as the number of trials per block, test stages, and presence of feedback.
